# Knockdown of long non-coding RNA plasmacytoma variant translocation 1 relieves ox-LDL-induced endothelial cell injury through regulating microRNA-30c-5p in atherosclerosis

**DOI:** 10.1080/21655979.2021.2019878

**Published:** 2022-01-18

**Authors:** Geng Li, Wenxia Zong, Lei Liu, Juan Wu, Jing Pang

**Affiliations:** a Department of Cardiology, Hubei No. 3 People’s Hospital of Jianghan University, Wuhan, China; b Department of Medical Laboratory, Eleven Wuhan Hospital, Wuhan, Hubei Province, China

**Keywords:** Atherosclerosis, long non-coding RNA plasmacytoma variant translocation 1, miR-30c-5p, inflammation, apoptosis, ox-LDL

## Abstract

Atherosclerosis (AS) is a chronic inflammatory disease involving endothelial dysfunction, and is one of the main causes of death from cardiovascular disease (CVD). Long non-coding RNA plasmacytoma variant translocation 1 (lncRNA PVT1) is overexpressed in the serum of CVD patients. However, the mechanism by which lncRNA PVT1 functions in AS remains unknown. Our research was designed to probe interactions involving lncRNA PVT1 and oxidized low-density lipoprotein (ox-LDL)-stimulated endothelial cell injury in AS. lncRNA PVT1 expression in the serum of AS patients and ox-LDL-stimulated human umbilical vein endothelial cells (HUVECs) was detected using reverse transcriptase quantitative polymerase chain reaction (RT-qPCR). Cell counting kit (CCK)-8 assays, flow cytometry (FCM), and enzyme-linked immunosorbent assay (ELISA) were used to determine cell proliferation, apoptosis, and levels of inflammatory cytokines, respectively. Moreover, the correlation between lncRNA PVT1 and miR-30 c-5p was predicted and verified using StarBase3.0, TargetScan, and luciferase reporter-gene assays. lncRNA PVT1 was overexpressed in the serum of AS patients and in ox-LDL-stimulated HUVECs relative to controls. Knockdown of lncRNA PVT1 facilitated proliferation, reduced apoptosis, and secretion of inflammatory factors in ox-LDL-treated HUVECs. Moreover, miR-30 c-5p was verified as a direct target of lncRNA PVT1. Furthermore, we observed that miR-30 c-5p expression was lower in AS patients than in controls. In addition, the influence of lncRNA PVT1 knockdown on ox-LDL-treated HUVECs was significantly reversed by downregulation of miR-30 c-5p. In conclusion, **l**ncRNA PVT1 silencing inhibited HUVEC damage stimulated by ox-LDL via miR-30 c-5p regulation.

## Introduction

Atherosclerosis (AS) is the most common and important arteriosclerosis vascular disease group and is characterized by the initiation of involved arterial lesions from the intima [1]. AS drives cardiovascular diseases (CVDs), which account for 17.9 million or 31% of all deaths worldwide each year [2]. Despite improvements in scientific invention, clinical research, and public health, the number of patients with AS is expected to continue to rise [3]. Therefore, new therapies are urgently needed to diagnose, prevent, and cure AS. AS is a complicated pathological process involving different types of cells and features, including angiogenesis, inflammatory responses, lipid metabolism, cell growth, and apoptosis [4,5]. These processes can result in myocardial infarction, ischemic stroke, and peripheral arterial disease [6]. Nevertheless, the roles of each event in AS remain unclear.

Multiple lncRNAs have been shown to play roles in AS and are implicated in regulating angiogenesis-associated element expression, endothelial cell viability, invasion, and tube formation, eventually influencing AS development [7–9]. Moreover, lncRNAs have been shown to be regulated in AS lesions, including endothelial cells (ECs), vascular smooth muscle cells (VSMCs), and macrophages [7]. lncRNA PVT1 has been found to be closely linked to human malignancies [10]. Domestic scholars have found that downregulation of lncRNA PVT1 expression suppresses the proliferation, migration, and phenotypic switching of human aortic smooth muscle cells (HASMCs) in aortic dissection (AD), providing a probable and latent therapy for AD [11]. A recent study reported that lncRNA PVT1 is upregulated in the serum of CVD patients, and is an independent risk factor in the progression of CVD, which identified the clinical significance of lncRNA PVT1 in CVD [4]. It has been shown that overexpression of lncRNA PVT1 promotes proliferative ability in atrial fibroblasts [12]. Additionally, lncRNA PVT1 can increase the level of ANGPT2 binding to VEGF to evoke AS, and the mechanism involved was assumed to be related to miR-26, but this remains to be confirmed [13]. Functional studies have suggested that lncRNA may act as endogenous molecular ‘sponges’ to affect the roles of various miRNAs and regulate their stability [9]. However, the molecular mechanisms by which lncRNAs and miRNAs mutually interact in AS need to be explored.

miRNAs have emerged as a new class of gene regulators that are crucial mediators in the setting of AS in various cells, including vascular and immune cells [14]. Endothelial cell injury is one of the first physiological changes in terms of pathophysiology, and results from inflammatory processes involving the endothelial monolayer of the vascular wall [15]. A variety of reports have revealed that ECs lining the entire vascular network are regarded as vital regulators in the development of AS [16]. Many classical risk factors involved in AS, such as ox-LDL, reactive oxygen species (**ROS**), Ang II, TNF-α, Hcy, and lipopolysaccharide (**LPS**), have been confirmed to result in EC apoptosis [17,18]. Among these, Ox-LDL is an important biomarker for the diagnosis of AS, which is involved in forming muscle-derived foam cells, and triggering a series of immune responses that may lead to AS [19]. High concentrations of ox-LDL enhance Fas-regulated EC apoptosis, and miRNAs regulate EC viability, apoptosis, and immune responses [20]. For instance, it has been reported that nuclear-enriched abundant transcript 1 (*NEAT1*) targets miR-30 c-5p, and upregulation of miR-30 c-5p reverses ox-LDL-stimulated effects in HUVECs [21]. Similarly, miR-30 c-5p is reported to be associated with NLRP3 inflammasome-modulated cell pyroptosis by targeting *FOXO3* in human aortic endothelial cells (HAECs) [22]. Therefore, we sought to illustrate the functions of lncRNA PVT1 in AS and demonstrated a link between lncRNA PVT1 and miRNAs. Functional analysis of miRNAs in ox-LDL-treated endothelial cell damage may provide a novel therapeutic avenue for AS treatment.

We hypothesized that lncRNA PVT1 might play a role in ox-LDL-induced endothelial cell damage via the regulation of miR-30 c-5p. Thus, our study was designed to determine whether lncRNA PVT1 affected HUVEC damage stimulated by ox-LDL via the regulation of miR-30 c-5p expression.

## Materials and methods

### Serum collection

Serum specimens were collected from 30 AS patients and 30 healthy individuals. These samples were immediately stored at −80°C. The characteristics of patients were presented in Table 1. Inclusion criteria: Patients aged over 18 years old, and patients with 50% stenosis in at least one coronary artery confirmed by invasive coronary angiography. Exclusion criteria: Patients with a history of hormone therapy in the last 3 months, and patients with intracranial hemorrhage, severe heart disease, stroke, vasculitis, nephrosis disease, liver disease, severe infections, thrombotic diseases and tumors. All participants in this serum collection signed a form providing written informed consent. This study was approved by the Ethics Committee of the Hubei No. 3 People’s Hospital of Jianghan University.

**Table 1.** The characteristics of patients

Parameters	Healthy control	AS patients	p value
Male/Female	15/15	15/15	–
Age (year)	44–75	45–73	>0.05
BMI	24 (18–25)	23(18–25)	>0.05
Hypertension	5(16.7%)	19 (63.3%)	<0.05
Diabetes mellitus (%)	7 (23.3%)	8(26.7%)	>0.05
Current smoke (%)	6 (20.0%)	9(30.0%)	>0.05
Biochemistry detection TG	1.32 ± 0.38	1.97 ± 0.69	<0.05
TC	3.41 ± 1.03	4.62 ± 1.57	<0.05
HDL-C	0.91 ± 0.36	1.28 ± 0.67	<0.05
LDL-C	2.75 ± 1.07	1.81 ± 0.61	<0.05

BMI, body mass index; TG, Triglyceride; TC, Total cholesterol; HDL-C, High-density lipoprotein cholesterol; LDL-C, Low-density lipoprotein cholesterol.

### Cell culture and treatment

HUVECs were obtained from American Type Culture Collection (Manassas, VA, USA) and cultured in F-12 K medium (Gibco, Grand Island, NY, USA) at 37°C under 95% humidity and 5% CO_2_. Specifically, supplementation with 10% fetal bovine serum (FBS; Gibco, Grand Island, NY, USA), 100 U/mL penicillin, and 100 μg/mL streptomycin (Gibco, Grand Island, NY, USA) was required. To simulate the primary stages of AS, HUVECs were induced by ox-LDL (Yiyuan Biotechnology, Guangzhou, China) at 100 μg/mL. After stimulation, the cells were subjected to the assays described below.

### Cell transfection

50 nM control-siRNA (GenePharma, Shanghai, China), 50 nM lncRNA PVT1-siRNA (GenePharma, Shanghai, China), 50 nM inhibitor control (cat. no. miR2N0000001-1-5; Guangzhou RiboBio Co., Ltd., Guangzhou, China), 50 nM miR-30 c-5p inhibitor (cat. no. miR20000244-1-5; Guangzhou RiboBio Co., Ltd., Guangzhou, China), 50 nM lncRNA PVT1-siRNA+50 nM inhibitor control, or 50 nM lncRNA PVT1-siRNA+50 nM miR-30 c-5p inhibitor was transfected into HUVECs using Lipofectamine 2000 (Invitrogen, Carlsbad, CA, USA) in accordance with the manufacturer’s instructions. At 24 h post-transfection, relative RNA levels were determined using RT-qPCR, and experiments were carried out subsequently.

### RT-qPCR analysis

Total RNA from serum and cells was isolated using TRIzol reagent (Invitrogen, Carlsbad, CA, USA) according to the manufacturer’s protocol. After that, 500 ng of extracted RNA was reverse-transcribed to cDNA using the PrimeScript RT Reagent Kit (TaKaRa, Beijing, China), and RT-qPCR analysis was conducted using the SYBR PrimeScript RT-PCR Kit (TaKaRa) using an ABI 7500 Real-Time PCR System (Applied Biosystem). Relative mRNA and miRNA expression levels were determined after normalization to *GAPDH* and small nuclear RNA *U6* as references and calculated using the 2-^ΔΔCt^ approach [23]. Primer sequences for PCR were listed as following:

U6 S, 5′-GGAACGATACAGAGAAGATTAGC-3′;

Stem-loop-R, 5′-CTCAACTGGTGTCGTGGAGTC-3′;

GAPDH forward, 5′-CATCATCCCTGCCTCTACTGG-3′;

reverse, 5′-GTGGGTGTCGCTGTTGAAGTC-3′;

Bcl-2 forward, 5′-AGGATTGTGGCCTTCTTTGAG-3′;

reverse, 5′-AGCCAGGAGAAATCAAACAGAG-3′;

Bax forward, 5′-TCTGAGCAGATCATGAAGACAGG-3′;

reverse, 5′-ATCCTCTGCAGCTCCATGTTAC-3′;

lncRNA PVT1 forward, 5′-TGAGAACTGTCCTTACGTGACC-3′;

reverse, 5′-AGAGCACCAAGACTGGCTCT-3′;

miR-30 c-5p forward, 5′-GCCGCTGTAAACATCCTACACT-3′;

reverse, 5′-GTGCAGGGTCCGAGGT-3′.

### CCK-8 assay [24]

Cell proliferation was assessed using the CCK-8 assay. HUVECs were plated overnight in 96-well plates. After that, the cells were transfected with control-siRNA, lncRNA PVT1-siRNA, lncRNA PVT1-siRNA+inhibitor control, or lncRNA PVT1-siRNA+miR-30 c-5p inhibitor at 37°C for 24 h followed by treating with 100 μg/mL ox-LDL for another 24 h. Then, 10  µL of CCK-8 reagent was added to each well and incubated for 4  h. The optical absorbance at 450 nm of the medium was measured using a microplate reader to determine cell proliferation.

### FCM analysis [25]

First, HUVECs were harvested with trypsin (Gibco) and washed with cold PBS (Gibco). Next, the cells were stained with Alexa Fluor® 488 Annexin V and propidium iodide (PI) kit (Invitrogen). Finally, the apoptotic cells (early+late apoptosis) were analyzed using a flow cytometer (BD Bioscience, San Diego, CA, USA).

### ELISA assay [26]

The release of inflammatory factors in HUVECs supernatant, including IL-6 (cat. no. ab178013), IL-1β (cat. no. ab214025), TNF-α (cat. no. ab181421), was assessed using ELISA kits (Abcam, Cambridge, MA, USA) following the manufacturer’s protocols.

### Dual luciferase reporter-gene assay [27]

StarBase3.0 were used to predict the binding sites between lncRNA PVT1 and miR-30 c-5p. The sequence of the wild-type (WT) 3ʹ-UTR of lncRNA PVT1, which contains binding sites for miR-30 c-5p, was cloned into pGL3 vector (Promega, Madison, WI, USA) to obtain a luciferase reporter-gene plasmid harboring lncRNA PVT1-WT. Mutant-type (MT) controls (lncRNA PVT1-MUT) were also constructed. Therefore, HUVECs were co-transfected with each plasmid and miR-30 c-5p mimic (cat. no. miR10000244-1-5; Guangzhou RiboBio Co., Ltd., Guangzhou, China) or mimic control (cat. no. miR1N0000001-1-5; Guangzhou RiboBio Co., Ltd., Guangzhou, China). Relative luciferase activity was determined using the Dual-Luciferase Reporter Assay System (Promega) following the manufacturer’s instructions.

### RNA pull-down assay [28]

After treatment, the correlation between miR-30 c-5p and lncRNA PVT1 was further confirmed using an RNA pull-down assay in accordance with the manufacturer’s instructions. Briefly, the biotinylated lncRNA PVT1 probe and oligo probe (Shanghai GenePharma Co., Ltd.) were incubated with M-280 Streptavidin magnetic beads (cat no. 60210; Invitrogen; Thermo Fisher Scientific, Inc.) according to the manufacturer’s protocol to obtain probe-coated beads. Then, the biotinylated RNA coated beads were separated. HUVECs cells (1x10^7^ cells) were harvested, lysed, sonicated and incubated with probe-coated beads at 4°C overnight. Finally, an RNA isolation kit (Thermo Fisher Scientific, Inc.) was used to extract the RNA complex bound to the beads, and the expression of miR-30 c-5p was analyzed by RT-qPCR.

### Western blotting [29]

Cellular proteins were extracted using Radio Immunoprecipitation Assay (RIPA) lysis buffer (Beyotime, Shanghai, China) and quantified using the Bicinchoninic acid (BCA) Protein Assay Kit (Beyotime). Next, 50 μg of proteins was loaded and separated on 12% or 15% gels by sodium dodecyl sulfate-polyacrylamide gel electrophoresis (SDS-PAGE), and proteins were transferred onto polyvinylidenefluoride (PVDF) membranes (Millipore, Billerica, MA, USA) at room temperature. The membranes were blocked in 5% skim milk in TBST for 1 h and incubated with primary antibodies against Bcl-2 (12789-1-AP, 1:1000, Proteintech, Wuhan, China), Bax (50599-2-lg, 1:1000, Proteintech), and GAPDH (ab9485, 1:2,500, Abcam) overnight at 4°C. The membranes were blocked with the appropriate anti-mouse IgG antibody (ab6728, 1:3000, Abcam) at room temperature for 1 h. Resultant bands were analyzed using an ECL chemiluminescence detection kit (Beyotime).

### Statistical analysis

Statistical analyses were conducted using SPSS v.20.0 (IBM Corp., Armonk, NY, USA). Statistically significant differences among groups were identified using unpaired Student’s *t*-test or one-way one-way analysis of variance (ANOVA) followed by Tukey’s post hoc test. All results are expressed as means ± SD from at least three independent experiments. P < 0.05 was considered to be statistically significant.

## Results

### lncRNA PVT1 was upregulated in serum from AS patients and ox-LDL-induced HUVECs

**Figure 1.**
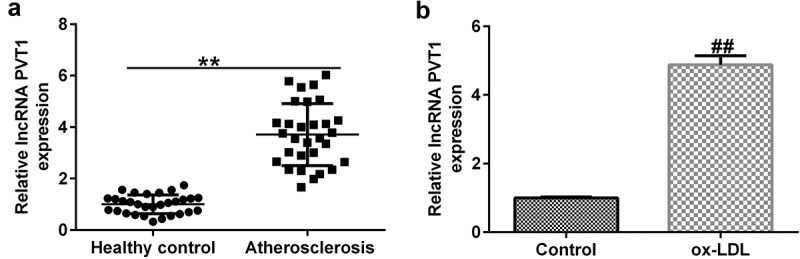
Expression of PVT1 in serum samples and HUVECs treated with ox-LDL by qRT-PCR. (a) High expression of PVT1 in serum samples. (b) Significantly high expression of PVT1 in HUVECs. **P < 0.01 vs. Healthy control; ^##^P < 0.01 vs. Control.

To illustrate the roles of lncRNA PVT1 in AS progression, serum samples were collected and HUVECs were exposed to ox-LDL. As presented in Figure 1(a), lncRNA PVT1 was upregulated in AS patients than in healthy individuals. The levels of lncRNA PVT1 were increased in HUVECs treated with 100 μg/mL ox-LDL (Figure 1(b)). These results indicated that lncRNA PVT1 was overexpressed in the serum of patients with AS and in ox-LDL-induced HUVECs.

### Roles of lncRNA PVT1-siRNA in HUVECs after ox-LDL stimulation

**Figure 2.**
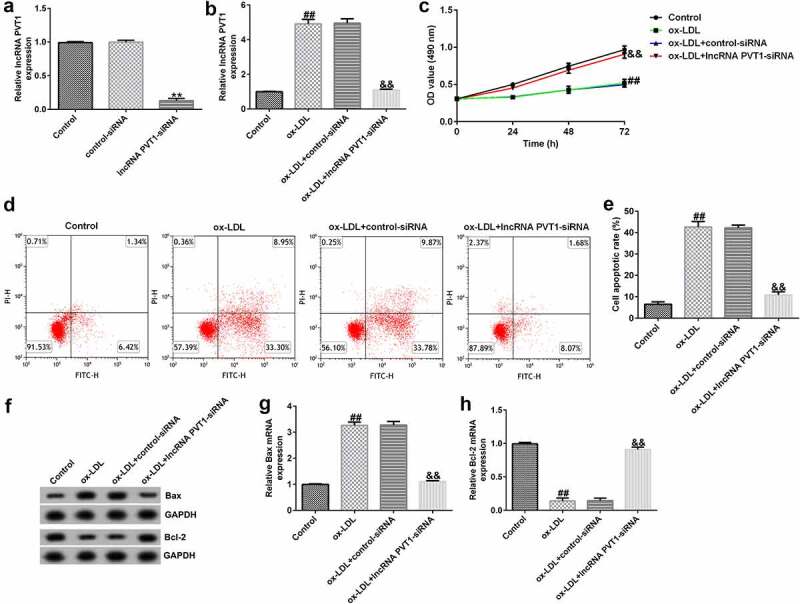
Knockdown of PVT1 facilitated proliferation in ox-LDL-induced HUVECs. HUVECs were transfected with control-siRNA or lncRNA PVT1-siRNA for 24 h, then exposed to ox-LDL for 24 h. (a and b) *PVT1* expression levels were measured via RT-qPCR. (c) The viability of HUVECs was assessed via CCK-8 assays after 24 h, 48 h, and 72 h. (d and e) Apoptotic cells were detected by FCM. (f) Western blot analysis of Bax and Bcl-2 expression. (g and h) RT-qPCR analysis of *Bax* and *Bcl-2* mRNA levels. **P < 0.01 vs. Control-siRNA; ^##^P < 0.01 vs. Control; &&P < 0.01 vs. ox-LDL+control-siRNA.

**Figure 3.**
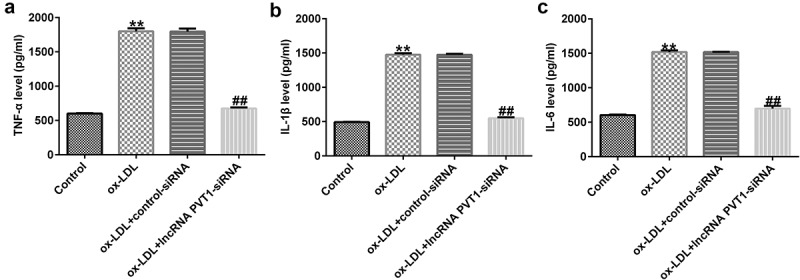
Inhibition of PVT1 suppressed inflammatory responses in ox-LDL-stimulated HUVECs. (a-c) TNF-α, IL-1β, and IL-6 expression was determined using ELISA. **P < 0.01 vs. Control; ^##^P < 0.01 vs. ox-LDL+control-siRNA.

To explore the function of lncRNA PVT1-siRNA in AS, HUVECs were transfected with control-siRNA and lncRNA PVT1-siRNA for 24 h, and exposed to ox-LDL for 24 h. Interference efficiencies were first assessed in HUVECs. RT-qPCR suggested that lncRNA PVT1-siRNA remarkably decreased the levels of lncRNA PVT1 compared to control siRNA (Figure 2(a)). The ox-LDL-induced enhancement in HUVECs was significantly inhibited by lncRNA PVT1-siRNA (Figure 2(b)). In contrast to those in the control siRNA group, the proliferation of HUVECs was drastically reduced (Figure 2(c)) and apoptosis (Figure 2(d,e)) was significantly increased in HUVECs after ox-LDL stimulation. In addition, protein and mRNA levels of Bax (an apoptosis-promoting marker) were enhanced (Figure 2(f,g)), while Bcl-2, an apoptosis inhibitor, and its cognate mRNA expression were decreased (Figure 2(f,h)) in ox-LDL-stimulated HUVECs. Moreover, ELISA results revealed that compared with the control group, ox-LDL treatment significantly enhanced the concentrations of TNF-α (Figure 3(a)), IL-1β (Figure 3(b)), and IL-6 (Figure 3(c)) in the supernatant of HUVECs, and these enhancements were significantly suppressed by lncRNA PVT1-siRNA. Therefore, these data illustrated that lncRNA PVT1-siRNA diminished ox-LDL-triggered inhibition of cell proliferation and decreased apoptosis.

### lncRNA PVT1 sponges miR-30 c-5p

**Figure 4.**
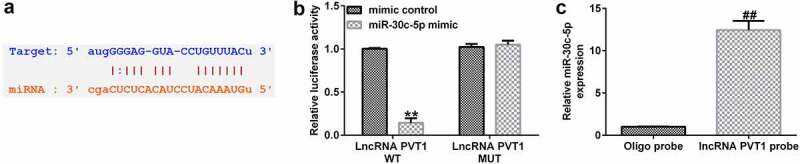
lncRNA PVT1 sponging of miR-30 c-5p. (a) miR-30 c-5p was identified as a latent target of lncRNA PVT1 via Starbase. (b) Detection of binding sites involving miR-30 c-5p and lncRNA PVT1 through dual-luciferase reporter-gene assays. (c) Detection of biotin-labeled miRNA in pull-down assays was performed. **P < 0.01 vs. mimic control; ^##^P < 0.01 vs. Oligo probe.

To understand the functional mechanisms involving lncRNA PVT1 in the process of AS, we used StarBase to predict that miR-30 c-5p binds competitively to lncRNA PVT1 (Figure 4(a)), and a correlation between miR-30 c-5p and lncRNA PVT1 was confirmed by dual-luciferase reporter-gene assays (Figure 4(b)). The results showed that compared to that of lncRNA PVT1-WT and mimic control groups, luciferase activity of lncRNA PVT1-WT was decreased, whereas no obvious differences were found in that of the lncRNA PVT1-MUT group. To further confirm whether lncRNA PVT1 sponges miR-30 c-5p, biotinylated miRNA pull-down assays were conducted. The results showed that lncRNA PVT1 probe significantly enhanced miR-30 c-5p expression compared to an oligo probe (Figure 4(c)). These data indicated that lncRNA PVT1 sponges miR-30 c-5p.

### miR-30 c-5p downregulation in AS patient serum and ox-LDL-induced HUVECs

**Figure 5.**
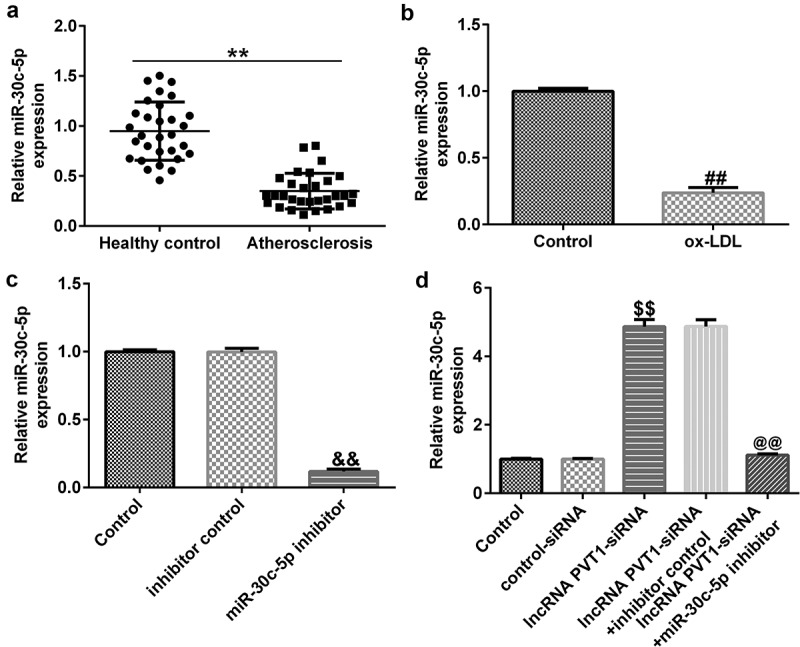
miR-30 c-5p expression in AS patient serum and ox-LDL-induced HUVECs. (a) Low expression of miR-30 c-5p in serum samples. (b) Significantly low expression of miR-30 c-5p in ox-LDL-induced HUVECs. (c) miR-30 c-5p expression in HUVECs transfected with inhibitor control or miR-30 c-5p inhibitor. (d) miR-30 c-5p expression in HUVECs transfected with control-siRNA, lncRNA PVT1-siRNA, lncRNA PVT1-siRNA+inhibitor control, or lncRNA PVT1-siRNA+miR-30 c-5p inhibitor.**P < 0.01 vs. Healthy control; ^##^P < 0.01 vs. Control; ^&&^P < 0.01 vs. inhibitor control; ^$$^P < 0.01 vs. Control-siRNA; ^@@^P < 0.01 vs. lncRNA PVT1-siRNA+inhibitor control.

To further investigate the role of miR-30 c-5p in AS progression, we determined the expression of miR-30 c-5p in serum from patients with AS and in ox-LDL-induced HUVECs. RT-qPCR analysis demonstrated that miR-30 c-5p levels were lower in the serum of patients with AS than in healthy individuals (Figure 5(a)). miR-30 c-5p was significantly downregulated in HUVECs treated with 100 *μ*g/mL ox-LDL than in controls (Figure 5(b)). Our findings indicated that miR-30 c-5p is downregulated in AS.

### lncRNA PVT1 negatively regulated miR-30 c-5p expression in HUVECs

The effects of lncRNA PVT1 and miR-30 c-5p expression on ox-LDL-treated HUVEC proliferation and apoptosis were analyzed. Control-siRNA, lncRNA PVT1-siRNA, inhibitor control, or miR-30 c-5p inhibitor was transfected into HUVECs for 24 h. As presented in Figure 5(c), miR-30 c-5p inhibitor significantly decreased miR-30 c-5p expression in HUVECs compared to the inhibitor control. lncRNA PVT1-siRNA significantly increased miR-30 c-5p levels in HUVECs compared to the control siRNA, and this was reversely inhibited by miR-30 c-5p inhibitor (Figure 5(d)).

### Protective role of lncRNA PVT1 knockdown in ox-LDL-stimulated HUVEC damage by miR-30 c-5p upregulation

**Figure 6.**
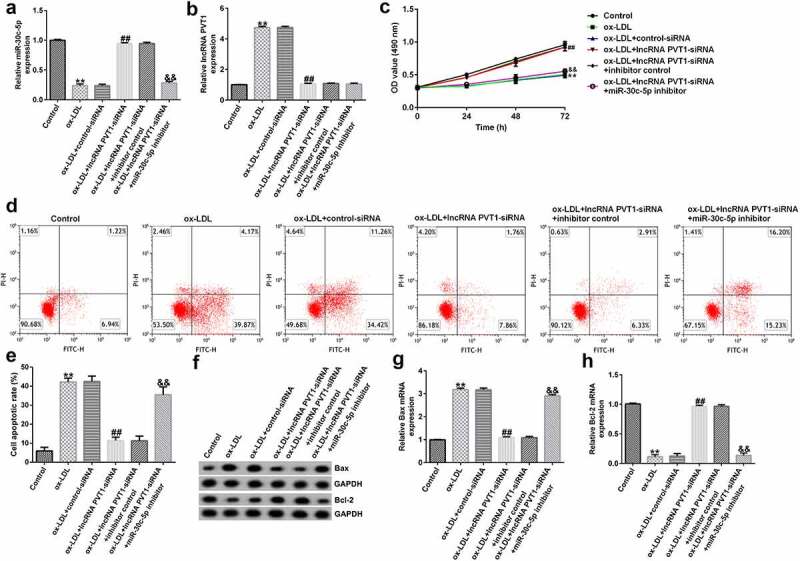
miR-30 c-5p relieved the negative effects on proliferation, apoptosis of ox-LDL-treated HUVECs by lncRNA PVT1-knockdown. HUVECs were transfected with control-siRNA, lncRNA PVT1-siRNA, inhibitor control, or miR-30 c-5p inhibitor for 24 h, then induced by ox-LDL for 24 h. (a) miR-30 c-5p expression in different groups. (b) lncRNA PVT1 expression in different groups. (c) Proliferation of HUVECs was assessed via CCK-8 assays after 24 h, 48 h, and 72 h. (d and e) Apoptotic cells were enumerated using FCM. (f) Western blot analysis of Bax and Bcl-2 expression. (g and h) RT-qPCR analysis of *Bax* and *Bcl-2* mRNA levels. **P < 0.01 vs. Control; ^##^P < 0.01 vs. ox-LDL+control-siRNA; ^&&^P < 0.01 vs. ox-LDL+lncRNA PVT1-siRNA+inhibitor control.

**Figure 7.**
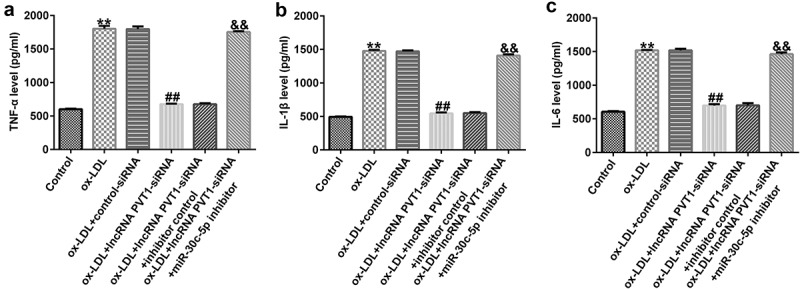
miR-30 c-5p relieved negative effects on inflammatory responses of ox-LDL-stimulated HUVECs by lncRNA PVT1 knockdown. (a-c) TNF-α, IL-1β, and IL-6 expression was assessed using ELISA. **P < 0.01 vs. Control; ^##^P < 0.01 vs. ox-LDL+control-siRNA; ^&&^P < 0.01 vs. ox-LDL+lncRNA PVT1-siRNA+inhibitor control.

To further demonstrate whether lncRNA PVT1 knockdown inhibited AS progression by regulating miR-30 c-5p, activity assays were performed. The results indicated that lncRNA PVT1-siRNA significantly enhanced miR-30 c-5p expression (Figure 6(a)), reduced lncRNA PVT1 expression (Figure 6(b)), increased cell proliferation (Figure 6(c)), reduced apoptosis (Figure 6(d,e)), decreased Bax mRNA and protein levels (Figure 6(f,g)), enhanced Bcl-2 expression (Figure 6(f,h)), and reduced the concentrations of inflammatory factors (Figure 7(a-c)) in ox-LDL-treated HUVECs compared to the ox-LDL group. It is worth mentioning that all these effects of lncRNA PVT1-siRNA on ox-LDL-treated HUVECs were reversed by miR-30 c-5p inhibitor. These results indicated that miR-30 c-5p inhibitor reversed the protective effects of relieving the impact of AS on HUVECs treated with ox-LDL exerted by lncRNA PVT1-siRNA.

## Discussion

This study determined whether lncRNA PVT1 affected HUVEC damage stimulated by ox-LDL via the regulation of miR-30 c-5p expression. The schematic diagram of summary of this study was presented in Supplementary Figure 1. The findings of present study indicated that lncRNA PVT1 gene silencing inhibited endothelial cell damage induced by ox-LDL by up-regulating miR-30 c-5p expression, suggesting a potential target for the treatment of AS.

AS is characterized by the formation of atherosclerotic plaques, endothelial injury, fat deposition, and the formation of a fibrous cap [30]. lncRNAs are closely associated with AS progression [31]. It has been reported that knockdown of lncRNA GAS5 reduces cell apoptosis, and this effect is reversed by miR-26a suppression in ox-LDL-treated human aortic ECs [32]. In addition, lncRNA MALAT1 is found to be downregulated by sacubitril/valsartan (S/V), which induces the inhibition of inflammation and apoptosis, as well as improvements in endothelial function in ox-LDL-treated HUVECs through inactivation of the TLR4/NF-κB signaling pathway [33]. A small number of well-researched lncRNAs have provided crucial clues regarding their potential therapeutic use in AS, while knowledge of mechanisms involving lncRNA PVT1 is still limited. lncRNA PVT1 is located in the chr8q24.21 region where there are targets with the highest DNA copy numbers in cancer cells, indicating a high risk of cancer [34]. Originally, lncRNA PVT1 was recognized as a normal retroviral integration site in murine leukemia virus-induced T lymphomas [35]. After deeper investigation, researchers have also revealed that lncRNA PVT1 is upregulated in various malignancies, such as cervical cancer, clear cell renal cell carcinoma, and thyroid cancer, and acts as an oncogene [36–38]. Additionally, upregulation of lncRNA PVT1 in serum derived from CVD patients was first confirmed in a recent study [4]. In addition, the level of lncRNA PVT1 is regarded as a risk factor for CVD by multivariate logistic regression testing [4]. The pathways mediating lncRNA PVT1 regulation of AS in vein endothelial cells (VECs) should be considered. In our study, lncRNA PVT1 overexpression in serum of patients with AS and in HUVECs treated with ox-LDL was ascertained. Then, a model of AS onset *in vitro* through treatment of HUVECs with ox-LDL was established to provide insight into the decisive behaviors of cell growth and apoptosis. The results showed that knockdown of lncRNA PVT1 facilitated cell proliferation in ox-LDL-induced HUVECs. Similar outcomes were achieved: downregulation of lncRNA PVT1 relieved ox-LDL-stimulated HUVEC injury and AS through the miR-153-3p/GRB2 axis via the ERK1/2 and p38 pathways [39]. Knockdown of lncRNA PVT1 (identified as a candidate diagnostic biomarker) directly increased the expression of miR‑190a‑5p in vascular endothelial cell proliferation to inhibit chronic heart failure (CHF) progression [40]. Therefore, the determination of lncRNA PVT1 levels is beneficial for understanding AS prognosis and treatment.

Because increasing evidence suggests that lncRNA PVT1 regulates AS development, the role of lncRNA PVT1 is largely uncharacterized functionally. LncRNAs can act as molecular miRNA sponges to affect the expression of target gene mRNAs, which has been one of the functions of lncRNAs identified in various biological activities [5]. Our research suggested that miR-30 c-5p is a potential target of lncRNA PVT1. Downregulation of miR-30 c-5p was found in the serum of AS and in ox-LDL-stimulated HUVECs. Similarly, in carotid AS (CAS), downregulation of lncRNA SNHG16 modulates the development of ox-LDL-stimulated CAS by upregulating the miR-30 c-5p/ADAM10 axis, suggesting a promising treatment strategy for CAS [41]. These results imply that the upregulation of miR-30 c-5p is significant for AS treatment. However, the mechanisms involved need to be elucidated. It is well known that low-density lipoproteins (LDL) and apolipoprotein B (apoB) are involved in the formation of AS, which is mainly caused by a disruption of lipid metabolism [42,43], and LDL levels in plasma have become a vital factor in assessing the risk of CVD and have therapeutic effects [44,45]. The accumulation of LDL induced activation of ECs, which could result in inflammatory responses, with macrophages absorbing altered lipoproteins and forming foam cells [46,47]. Studies have shown that very low-density lipoprotein (VLDL) levels are reduced because microsomal triglyceride transfer protein is inhibited by miR-30 c overexpression [45,47,48]. Thus, enhanced miR-30 c-5p levels could be used as promising candidates for AS treatment.

The present study revealed that lncRNA PVT1 sponges miR-30 c-5p and negatively regulates miR-30 c-5p levels in HUVECs. Previous studies have confirmed the function of lncRNAs on miRNAs [49]. For example, lncRNA SNHG12 is highly expressed in AS, promotes pro-inflammatory factor secretion, and enhances atherosclerotic lesions *in vivo*. SNHG12 knockdown effectively suppresses ox-LDL-stimulated injury to HUVECs through depletion of miR-218-5p mediated by insulin growth-like factor 2 (IGF2) [50]. Moreover, miR-199a-5p was also validated as a target of SNHG12 in that SHNG12 participates in the pathophysiological process of AS progression by targeting miR-199a-5p/HIF-1α [51]. MALAT1, which induces autophagy in ECs, downregulation in brain microvascular endothelial cells (BMECs) may contribute to inflammation suppression and AS by regulating the miR-200 c-3p/ SIRT-1 axis [52]. Consistent with these findings, we investigated the functions of lncRNA PVT1-knockdown in ox-LDL-stimulated HUVEC damage through upregulation of miR-30 c-5p. We found that lncRNA PVT1-knockdown intensified the protective roles in ox-LDL-treated HUVEC damage, and these effects were reversed by miR-30 c-5p suppression. Based on these discoveries, we can conclude that functional miRNAs are of high priority for EC senescence and dysfunction in AS [53]. AS development involves complicated cross-talk between vascular cells (endothelial and smooth muscle cells) and immune cells, where miRNAs are key regulators of gene expression at the post-transcriptional level [54]. Collectively, in our current study, miR-30 c-5p was upregulated in ox-LDL-stimulated HUVECs, which is related to its anti-inflammatory and anti-atherosclerotic effects *in vitro* by knockdown of lncRNA PVT1. In our investigations, interactions involving miR-30 c-5p and lncRNA PVT1 were confirmed in HUVECs, suggesting that lncRNA PVT1/miR-30 c-5p may be developed as a new therapeutic pathway involved in AS.

In order to make the role and significance of lncRNA PVT1 and miR-30 c-5p in AS more convincing, more in-depth exploration is needed. For example, the correlation between the expression of lncRNA PVT1/miR-30 c-5p in AS patients and the pathological parameters of AS patients needs to be clarified. Besides, the role and mechanism of lncRNA PVT1/miR-30 c-5p in AS should be investigated in animal models. We will continue to conduct in-depth research on these issues in the next step of research.

## Conclusion

In conclusion, the present study highlighted that knockdown of lncRNA PVT1 protected ox-LDL-treated HUVECs by miR-30 c-5p upregulation, promoting cell growth, and inhibiting apoptosis. Therefore, the lncRNA PVT1/miR-30 c-5p axis can be distinguished as a new molecular mechanism and potential therapeutic tool for AS treatment. Notably, our observations provide a rationale for targeting lncRNA PVT1 to treat AS abnormality-associated diseases.

## Supplementary Material

Supplemental Material

## Data Availability

All datasets used and/or generated during the current study are available from the corresponding author upon reasonable request.

## Research highlights

- lncRNA PVT1 was upregulated in serum from AS patients and ox-LDL-induced HUVECs;
- lncRNA PVT1-siRNA diminished ox-LDL-triggered inhibition of cell proliferation and decreased apoptosis in HUVECs;
- lncRNA PVT1 knockdown played a protective role in ox-LDL-stimulated HUVEC damage by miR-30c-5p upregulation
