# Supplementary figures and images for: Knockdown of long non-coding RNA plasmacytoma variant translocation 1 relieves ox-LDL-induced endothelial cell injury through regulating microRNA-30c-5p in atherosclerosis
Source: Bioengineered. 2022 Jan 18;13(2):2791–802. doi: 10.1080/21655979.2021.2019878 (PMC8974020; doi:10.1080/21655979.2021.2019878)

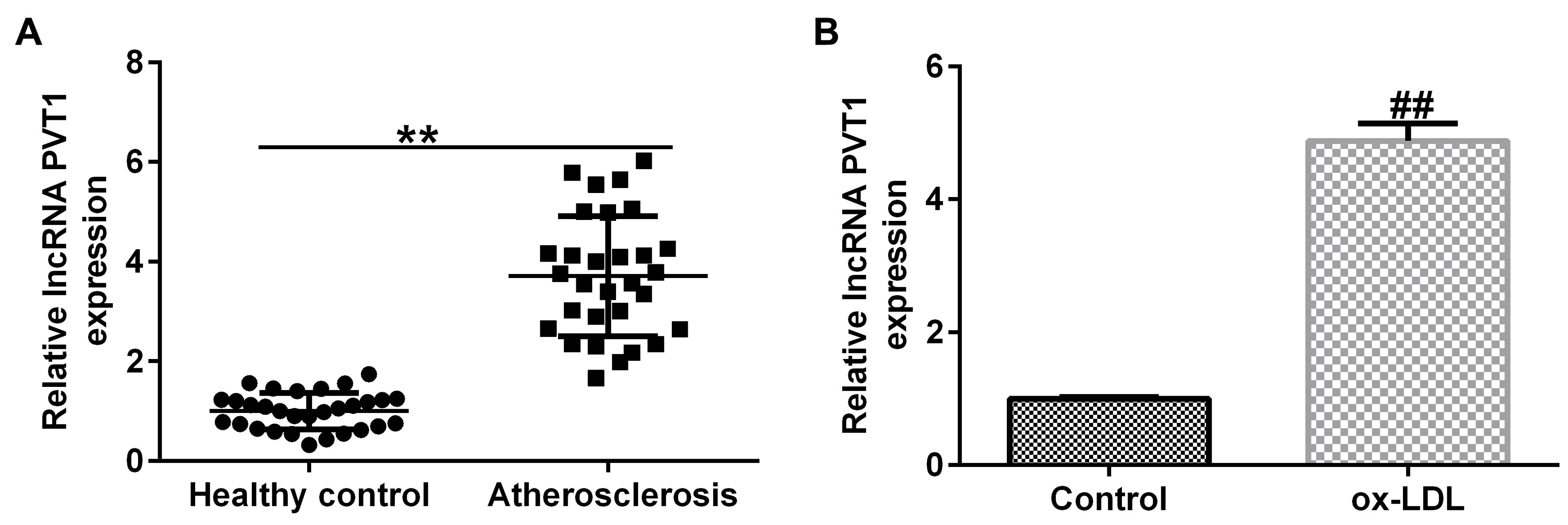

Supplement: Supplemental Material [file KBIE_A_2019878_SM7643.zip › supplementary/Supplementary Figure 1.tif]
